# Developing and evaluating interventions to reduce inappropriate prescribing by general practitioners of antibiotics for upper respiratory tract infections: A randomised controlled trial to compare paper-based and web-based modelling experiments

**DOI:** 10.1186/1748-5908-6-16

**Published:** 2011-03-03

**Authors:** Shaun Treweek, Ian W Ricketts, Jillian Francis, Martin Eccles, Debbie Bonetti, Nigel B Pitts, Graeme MacLennan, Frank Sullivan, Claire Jones, Mark Weal, Karen Barnett

**Affiliations:** 1 Quality, Safety and Informatics Research Group, University of Dundee, Kirsty Semple Way, Dundee, UK; 2 School of Computing, University of Dundee, Queen Mother Building, Dundee, UK; 3 Health Services Research Unit, University of Aberdeen, Health Sciences Building, Foresthill, Aberdeen, UK; 4 Institute of Health and Society, University of Newcastle, Baddiley-Clark Building, Richardson Road, Newcastle upon Tyne, UK; 5 Clinical and Population Science and Education, University of Dundee, Kirsty Semple Way, Dundee, UK; 6 School of Electronics and Computer Science, University of Southampton, Southampton, UK

## Abstract

**Background:**

Much implementation research is focused on full-scale trials with little evidence of preceding modelling work. The Medical Research Council Framework for developing and evaluating complex interventions has argued for more and better theoretical and exploratory work prior to a trial as a means of improving intervention development. Intervention modelling experiments (IMEs) are a way of exploring and refining an intervention before moving to a full-scale trial. They do this by delivering key elements of the intervention in a simulation that approximates clinical practice by, for example, presenting general practitioners (GPs) with a clinical scenario about making a treatment decision.

**Methods:**

The current proposal will run a full, web-based IME involving 250 GPs that will advance the methodology of IMEs by directly comparing results with an earlier paper-based IME. Moreover, the web-based IME will evaluate an intervention that can be put into a full-scale trial that aims to reduce antibiotic prescribing for upper respiratory tract infections in primary care. The study will also include a trial of email versus postal invitations to participate.

**Discussion:**

More effective behaviour change interventions are needed and this study will develop one such intervention and a system to model and test future interventions. This system will be applicable to any situation in the National Health Service where behaviour needs to be modified, including interventions aimed directly at the public.

**Trial registration:**

ClinicalTrials (NCT): NCT01206738

## Background

Although much is known about interventions that can be effective in changing the behaviour of health professionals [1], the literature provides little information to guide the choice, or to optimise the components, of these interventions [2,3]. Implementation research is the scientific study of methods to promote the uptake of research findings to improve the quality of care. However, much implementation research is focused on full-scale trials with little evidence of preceding modelling work. The Medical Research Council (MRC) framework for developing and evaluating complex interventions has argued for more and better theoretical and exploratory work prior to a trial as a means of improving intervention development [4].

The use of intervention modelling experiments (IMEs) for interventions that aim to change behaviour is one approach to doing exploratory work [5]. In an IME key elements of the intervention are delivered (generally in a randomised controlled trial, hence use of the word 'experiment') in a manner that approximates the real world but the measured outcome is generally an interim outcome, a proxy for the clinical behaviour of interest (clinical decision in response to a simulated clinical encounter) prior to entering the intervention into a full-scale trial. To date IMEs have been paper-based [5,6] but this may limit their efficiency, acceptability and ecological validity. Web-based IMEs have the potential to provide much richer simulations of clinical encounters and allow measurement of key process variables such as time to make a decision. The current study will develop a web-based IME system based around an existing web-based intervention delivery system called Lifeguide http://www.lifeguideonline.org/[7]. We will then use the new system to run a web-based IME on antibiotic prescribing for upper respiratory tract infections (URTIs) in general practice.

Cochrane reviews of the effectiveness of antibiotics in treating URTIs suggest no benefit in colds [8] and only marginal benefit for uncomplicated sore throat [9]. A large cohort study of the use of antibiotics for respiratory tract infection in UK primary care concluded that antibiotics are not justified to reduce the risk of serious complications in URTIs [10]. Primary care accounts for 80% of total antimicrobial use and 60% of that is for respiratory infections, which are on the whole self-limiting [11]. The web-based IME will systematically develop and evaluate theory-based interventions for reducing unnecessary prescribing of antibiotics that correspond to the theoretical, modelling and experimental phases of the MRC framework. We will directly compare this work with a previously published paper-based IME on the same topic [6], allowing us to evaluate the two methods of developing and simulating interventions prior to a full-scale trial. We propose that the most effective intervention will go on to be evaluated in a separately funded full-scale trial.

### Trial objectives

1. To run a web-based IME to develop and evaluate theory-based interventions to reduce antibiotic prescribing for URTIs in primary care.

2. To compare the effectiveness of email and postal invitations to general practitioners (GPs) to take part in the web-based IME in a randomised controlled trial.

3. To compare the web-based and paper-based methods of running IMEs in terms of identification of predictors, the type of intervention that can be simulated, and the effect on intended prescribing behaviour on trial conduct of an IME.

## Methods

### Trial design

#### General

The study will include two trials, one a web-based IME, the other an evaluation of the method used to invite GPs to take part in the web-based IME. The basic design of these two trials will be:

1. Web-based IME: a three-arm, double-blind, randomised controlled trial of two behaviour change interventions compared to generic information.

2. Invitation: a two-arm, randomised controlled trial of an email invitation to GPs compared to a postal invitation.

The web-based IME will be based on a previous paper-based IME [6]. This earlier work provided qualitative data about the kind of attitudes and beliefs among GPs about the use of antibiotics in the treatment of URTIs (cough/cold, sore throat/tonsillitis in patients of any age). This was used to develop questionnaire items relevant to the clinical context of treating URTIs in primary care and which operationalised the constructs of our chosen theories (*e.g*., theory of planned behaviour: questionnaire has an item asking GPs whether they thought patients expected them to prescribe an antibiotic, which is linked to the theory's subjective norms construct). The earlier work also developed clinical scenarios describing patients presenting in primary care with symptoms of URTI. GPs were asked to report their clinical decisions in response to the scenarios and these were used to generate scores for 'simulated behaviour.' We will use a web-based IME to deliver the questionnaire and scenarios to GPs in order to identify predictors of prescribing behaviour and compare these with those identified in the paper-based IME.

**Figure 1:**
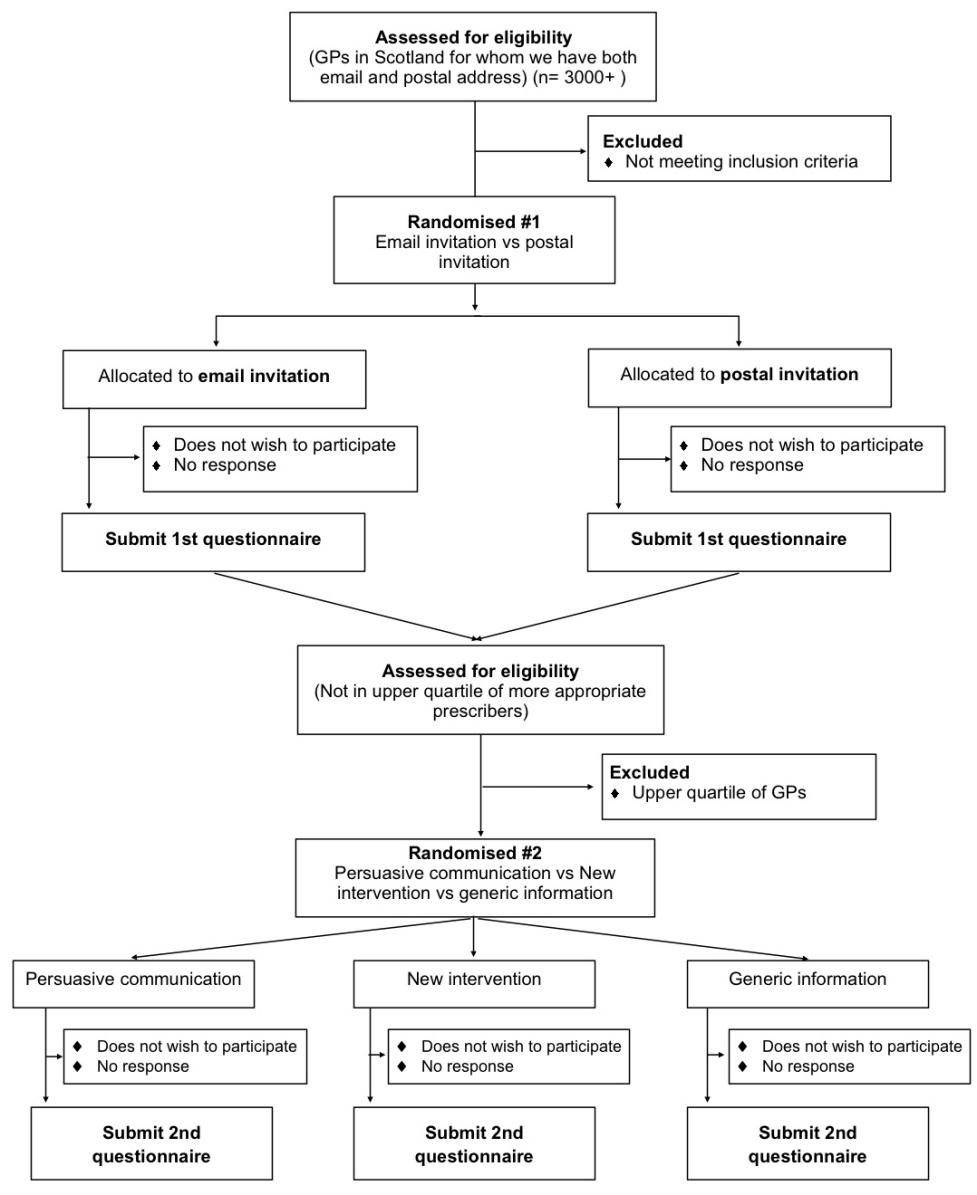
**Flow diagram - Randomised controlled web-based IME with an embedded trial of email versus postal invitation to participate**.

The research study is based in the Division for Clinical and Population Sciences and Education at the University of Dundee at Ninewells Hospital, with support from the Universities of Aberdeen, Newcastle and Southampton and the Scottish School of Primary Care. It is funded by the Chief Scientist Office, grant number CZH/4/610 and approved by the Tayside Committee on Medical Research Ethics A, REC reference 10/S1401/54. A flow diagram of the study is shown in Figure 1.

#### Participants

Inclusion criterion is GPs in Scotland. Exclusion criterion is the inability to obtain both an email address and a postal address for the GP.

#### Identifying participants

GPs will be identified by the Scottish Primary Care Research Network (SPCRN; http://www.sspc.ac.uk/spcrn/) [12] using a combination of publicly available information provided by ISD Scotland http://www.isdscotland.org/isd/3793.html[13] and restricted information held on the NHS.net database. A preliminary test of our recruitment method has identified over 3,000 GPs for whom we can obtain both email and postal addresses.

GPs receiving a postal invitation will receive a one-page letter and a two-page information sheet. Together with general information, the letter will contain a URL to the web-based IME system and a unique identifier for the GP. The identifier will be used to identify non-responders and for payment (see below). GPs receiving an email invitation will receive an email containing the same text as on the paper letter and the two-page information sheet as a pdf attachment. We will send two reminders to non-responders, the first at two weeks, the second at four weeks, using the same method as used for the initial invitation. We expect response rates of 30% to 40% based on the earlier paper-based IME [6].

Participants will be asked to complete up to two online questionnaires, which together will form a web-based IME. GPs will be paid £20 in advance in the form of a voucher for the first part of the web-based IME and £30 in advance as a voucher for the second part. A recent Cochrane review of strategies to increase response rates to postal and electronic questionnaires found that advanced payment led to a higher response rate (odds ratio = 2.00; 95% confidence interval = 1.54 to 2.60) [14]. GPs will be able to select from a list of seven types of voucher: Amazon, Argos, Boots, iTunes, Love2Shop, Marks & Spencer, or Starbucks. GPs will also be free to do the study without receiving a voucher if they so wish.

GPs will be able to decline to take part in the study by clicking a button on the second page of the web-based IME system, by emailing their ID number to a study email address, or by telephoning or sending a text message to the study mobile number. A flow diagram of the study is shown in Figure 1.

#### Informed consent

GPs will not be expected to provide explicit consent; a completed questionnaire will be taken as implied consent.

#### Randomisation

The study statistician will generate a list of random numbers to be used by SPCRN to randomly allocate GPs to receive either an email or postal invitation on a 1:1 basis. There will be no stratification. The list will be generated using Stata 11.0 statistical software (StataCorp, College Station TX). To ensure we do not approach too many GPs to participate in the main study we will randomise two initial blocks of 250 GPs (see Sample size) then subsequent blocks of 50 GPs will be contacted until the required sample size for the web-based IME is met.

All GPs will complete the same, initial web-based questionnaire to identify predictors of behavioural intention and simulated behaviour. GPs will receive one of two interventions (one of which will be a persuasive communication) or a comparator with the second web-based questionnaire. The study statistician will generate a list of random numbers for allocation to intervention or comparator on a 1:1:1 basis, which will be loaded into the web-based IME system. Allocation will, therefore, be done by the system when the GP logs onto the web-based IME system to complete the second questionnaire. There will be no stratification.

#### Blinding

Study participants, the trialists, and the trial statistician will be blind to allocation. Staff at the SPCRN will know the allocation for the email versus postal invitation study but not the web-based IME intervention study.

#### Trial procedures

Participants will be asked to complete up to two online questionnaires, which together will form a web-based IME. GPs who are already likely to be following best evidence with regard to prescribing antibiotics will be excluded from the second questionnaire, which we will do by excluding those in the first quartile of 'intention to prescribe' responses to the first questionnaire. These 25% of GPs will be thanked but will not continue in the study. Thus 75% of GPs completing the first questionnaire will be asked to complete the second. The intervention will be delivered together with the second questionnaire.

#### Duration

Participants will remain in the study until they complete the second questionnaire, which we expect to be between four and five months after they receive the invitation to take part.

### Interventions and comparator

#### Interventions

The web-based IME will deliver one of the interventions used in the paper-based IME [6] -- a persuasive communication -- and a second, new intervention developed for the web-based IME. The persuasive intervention aimed to reinforce the GP's beliefs about the positive consequences of managing sore throat without prescribing antibiotics. The materials for the persuasive communication can be downloaded at http://www.biomedcentral.com/content/supplementary/1472-6963-8-11-s2.doc[15]. This intervention did change GPs' beliefs in the paper-based IME, and we aim to see if this is replicated in the web-based IME.

However, we will also deliver a new intervention that takes advantage of the possibilities of web delivery. This will allow a comparison between an intervention simulated using web and paper-based methods and, additionally, provide a head-to-head comparison between that intervention and an intervention developed without the constraints of paper delivery.

The new intervention, designed to take advantage of the possibilities offered by web-based delivery, will be designed using predictors identified from the first questionnaire and by using appropriate behaviour change techniques [16,17]. There is no widely agreed taxonomy by which to specify methods of delivery of behaviour change techniques; we will use as a starting point the suggestions of the Evidence-based Behavioural Medicine Committee (EBMC) [18]. Suggested dimensions are: provider, format, setting, recipient, duration, and frequency. We will use the EBMC suggestions to map the effectiveness of the interventions in the primary studies included in the systematic reviews populating it with the delivery methods used in the studies in the systematic reviews of strategies to decrease antibiotic prescribing [19,20] and the results from the first questionnaire. We will send the results of the mapping exercise to a small number of experts for external validation.

We will use 'intervention mapping' [21] to systematically consider combinations of content and methods of delivery; there are likely to be logical combinations. By considering such combinations we will produce a range of potential new interventions.

We will pilot each of the potential new interventions (up to three) with groups of three to six GPs. Whilst delivering the intervention we will ask them to 'think aloud' about their reactions to the intervention, which allows us to identify problems and fine-tune content and delivery. We will select the most promising intervention for evaluation in the web-based IME. GPs involved in developing the intervention will not be included in the web-based IME.

#### Comparator

The comparator will be provision of general information about prescribing of antibiotics taken from recent reviews on antibiotic prescribing [8,9].

#### Outcome measures

For the web-based IME, the primary outcomes will be behavioural intention and behavioural simulation, which have been shown to be reliably related to behaviour [16,22]. Three questions will assess GPs' strength of intention to manage URTIs without antibiotics (*e.g*., over the next 12 weeks, when a patient presents with a URTI, I have in mind to manage them without prescribing an antibiotic (rated on 7-point Likert scales 'Strongly Disagree' - 'Strongly Agree'). Responses will be summed with a low score corresponding to a low intention to prescribe antibiotics. Sixteen clinical scenarios from the paper-based IME work will provide the materials to measure simulated behaviour (eight in the first questionnaire, eight more in the second). Respondents will be asked how they will manage the patients in the scenarios and asked to rate, on a score out of 10, the difficulty of making their clinical decision.

Secondary outcomes are concerned with evaluating the relative utility of web-based and paper-based IMEs. For this, we will compare the following outcomes for each method.

##### 1. Behaviour change techniques that can be operationalised

We will develop a matrix of behaviour change techniques that can be operationalised by each IME method. This work will be done with researchers (especially health psychologists) unconnected with the project in brainstorming workshops. Project team members will present work done using paper-based and web-based IMEs, and participants will work alone to place behaviour change techniques into the matrix. Following this group discussion will be used to reach consensus on which techniques could be supported by each method. We will do this with more than one group of researchers (*e.g*., at weekly departmental research meetings at collaborator sites). Here we are looking for the method that can be to deliver most behaviour change techniques.

##### 2. Complexity of scenarios that can be delivered

We will develop a table of factors (*e.g*., people involved, context) that we are able to vary in clinical scenarios that can be delivered by each IME method. We will use brainstorming workshops involving researchers unconnected to the project to validate this list (*i.e*., do they agree with it) and make suggestions as to whether it can be extended (or reduced) for each IME method. We may use the same groups of researchers as for point one above although not at the same workshop. Here we are looking for the method that would allow more factors of a scenario to be varied.

##### 3. Identification of predictors

The first part of the web-based IME will evaluate whether the delivery mechanism of the IME (paper or web) affects predictors of GP behaviour. We anticipate that the predictors coming from the two methods will be similar because only the delivery method will be different; the web-based IME will use materials from the earlier paper-based IME. For example, evidence of habitual behaviour was strongly correlated with behavioural intention in the earlier paper-based IME. When delivering the same scenarios to GPs in a web-based IME, we anticipate (but do not know) that we will also find a strong correlation between habit and intended behaviour. Therefore, here we are seeking confirmation that delivery method alone does not lead to widely different predictors being identified by the IME.

##### 4. Recruitment (reaching target and time taken to do so)

Here, we will assess three things: time (in days) spent by the project team to recruit to target; time (in days) between sending an invitation to a potential participant and receiving a completed questionnaire; and the number of invitations necessary to receive one completed questionnaire

These will be measured in the first part of the web-based IME and compared to estimates of timing (admittedly, less accurate) from the earlier paper-based IME. We will, however, get an indication of how the two methods compare. The embedded trial of paper invitation to GPs to participate in our study versus email invitation will provide comparative data on these two recruitment methods, which will be of wider relevance than recruitment to IMEs. Here we are looking for the fastest and least labour-intense method of recruiting participants to an IME.

##### 5. Ability to change targeted constructs

The theories used in paper- and web-based IMEs target particular constructs (*e.g*., GPs' beliefs about antibiotic prescribing) and the effect an intervention has on these targets is measured using a score. The web-based IME will involve a direct comparison of an intervention from the paper-based IME and a new intervention that makes use of the possibilities offered by web-based delivery. We will also compare the effect on targeted constructs of the same persuasive communication intervention delivered by both methods. By comparing each IME method's ability to change scores on the targeted constructs we will be able to make a statement as to which IME method is most effective.

##### 6. Effect size for persuasive communication intervention compared to control

The persuasive communication intervention and control were delivered in an earlier paper-based IME. By delivering the same intervention in a web-based IME we can compare the effect on the primary outcome (intention to prescribe an antibiotic) of the two delivery methods. Here, we are looking for the greatest effect size.

##### 7. Qualitative work: ease of administration and GP feedback

Running an IME is not trivial and a method that provides some administrative benefits (*e.g*., easier to put scenarios together, easier to pilot scenarios with some participants, easier to change the order of scenarios, easier to deliver to participants, easier to manage collected data) has efficiency advantages. We will compare, via interviews, experiences of the research team with running paper-based and web-based IMEs. This will, of course, be subjective (although item four will provide some quantitative information about effort linked to recruitment) but will nevertheless provide useful information about the effort required by researchers to run an IME via each approach. Here, we are looking for the method that is considered easiest to use and which uses fewest resources for an IME.

We will also consider qualitative feedback from GPs on the web-based delivery method and the utility of decision time data collected in the web-based IME, which is impossible to measure with paper-based IMEs. This work will also consider how the differing configurations of the interventions might explain their effects. Here, we are looking for feedback from GPs that identifies whether they think web-based IMEs have face validity, that the approach is feasible, and that they would participate in future web-based IMEs.

For the email versus postal invitation trial, the primary outcome is the number of GPs completing the first questionnaire. There are no secondary outcomes.

### Safety

No safety issues or potential adverse events linked to participation are envisaged.

### Statistics

#### Sample size

Our sample size will be 250 GPs completing the first questionnaire, 75% of whom will be invited to complete the second questionnaire. GPs who are already likely to be following best evidence with regard to prescribing antibiotics will be excluded from the second questionnaire, which we will do by excluding those in the first quartile of 'intention to prescribe' responses to the first questionnaire. These 25% of GPs will be thanked but will not continue in the study. For the first questionnaire analysis, our required sample size will be 162 based on a recommendation by Green [23] for multiple regression analysis with 14 predictor variables. The analysis of the questionnaire data will check the internal consistency of the measures and use multiple regression analysis to examine the relationships between predictor and outcome variables.

Using the dependent variable of behavioural intention, we will seek to detect an effect size of 0.66. This was the mean effect size for change in intention in a meta-analysis of trials that measured change in intention and behaviour [24]. This 'medium to large' change in intention led to a 'small to medium' change in behaviour. We will require 50 participants per group to have 90% power of detecting this effect size at a significance level of 5% to 150 participants in total. We anticipate having 187 GPs in our 75% sample from the first questionnaire study, which will allow for some dropout.

The effect of each intervention will be estimated by comparing the change in intention in each of the active intervention groups with that in the control group. This procedure will be repeated with behavioural simulation as the dependent variable. We propose to take the intervention with the largest effect size forward into a definitive trial; there is a risk of wrongly choosing this as the most effective (a type 3 error). With 50 practitioners per group if treatment B is actually superior to treatment A, such that the standardised difference in mean scores is 0.38, the probability of incorrectly choosing treatment A is 5%.

### Statistical analysis

For the web-based IME trial, reporting will be in line with the CONSORT statement [25]. Data will be summarised by intervention group using appropriate descriptive statistics where required, for example, mean and standard deviation for the primary outcomes. The primary outcomes will be analysed using an analysis of covariance (ANCOVA) framework to correct for the baseline levels of intention and behavioural simulation. Effect sizes will presented with estimated 95% confidence intervals of the two new interventions versus generic information as the comparator. All analysis will be by intention to treat. Missing data at baseline are impossible by trial design, missing outcome data due to non-response to second questionnaire will not be imputed, analyses will be by responders only. Characteristics of responders and non-responders baseline data will be compared to assess the impact potential non-response on results. Secondary outcome will be analysed in a similar manner to the primary outcomes.

For the email versus postal invitation trial, reporting will also be in line with the CONSORT statement [25]. The primary outcome will be presented as the proportion of GPs sent an invitation completing the first questionnaire by invitation method. Proportions will be compared using a simple chi-square test, and results presented as an odds ratio and 95% confidence interval. There are no secondary analyses for this trial.

## Discussion

The National Health Service and other healthcare providers need effective quality improvement interventions to be put into clinical practice, which requires effective behaviour change interventions. IMEs are a way of exploring and refining an intervention before moving to a full-scale trial as recommended in the MRC framework for evaluating complex interventions. IMEs do this by delivering key elements of the intervention in a simulation that approximates clinical practice by, for example, presenting GPs with a clinical scenario about making a treatment decision. Earlier IMEs have been paper-based, which limits what can be done in the simulation.

Web-based IMEs provide the potential for better clinical simulations, which can be expected to lead to better interventions. The study described here will run a full, web-based IME involving 250 GPs that will advance the methodology of IMEs by directly comparing results with an earlier paper-based IME. Moreover, the web-based IME will evaluate an intervention that can be put into a full-scale trial that aims to reduce antibiotic prescribing in primary care. Reducing inappropriate prescribing of antibiotics in general practice is a national priority; indeed, antibiotic use is increasing in the UK and Scotland's prescribing is second highest amongst UK administrations. More effective behaviour change interventions are needed, and this proposal will develop one such intervention and a system to model and test future interventions. This system will be applicable to any situation in a healthcare system where behaviour needs to be modified, including interventions aimed directly at the public.

We believe the web-based IME system will reduce the overall cost of trials by requiring fewer iterations of full-scale trial/analysis/revisions/full-scale trial before an optimised intervention is produced, thereby making better use of limited research budgets for trials. Finally, a web-based system can involve a wide range of stakeholders, expanding the range of opinions that can feed into intervention design and development, and enhancing the implementation potential of quality improvement initiatives.

## Competing interests

Martin Eccles is Co-Editor in Chief of Implementation Science; all decisions on this manuscript were made by another editor.

## Authors' contributions

All authors contributed to the design of the study. ST wrote the first draft of the protocol and all authors contributed to the final version. All authors have approved the final manuscript.
